# α‐Synuclein V15A Variant in Familial Parkinson's Disease Exhibits a Weaker Lipid‐Binding Property

**DOI:** 10.1002/mds.29162

**Published:** 2022-07-27

**Authors:** Kensuke Daida, Shotaro Shimonaka, Kahori Shiba‐Fukushima, Jun Ogata, Hiroyo Yoshino, Ayami Okuzumi, Taku Hatano, Yumiko Motoi, Tomoki Hirunagi, Masahisa Katsuno, Hideo Shindou, Manabu Funayama, Kenya Nishioka, Nobutaka Hattori, Yuzuru Imai

**Affiliations:** ^1^ Department of Neurology Juntendo University School of Medicine Tokyo Japan; ^2^ Department of Diagnosis, Prevention, and Treatment of Dementia Juntendo University Graduate School of Medicine Tokyo Japan; ^3^ Research Institute for Diseases of Old Age Juntendo University Graduate School of Medicine Tokyo Japan; ^4^ Department of Drug Development for Parkinson's Disease Juntendo University Graduate School of Medicine Tokyo Japan; ^5^ Department of Research for Parkinson's Disease Juntendo University Graduate School of Medicine Tokyo Japan; ^6^ Department of Neurology Nagoya University Graduate School of Medicine Nagoya Japan; ^7^ Department of Lipid Signaling National Center for Global Health and Medicine Tokyo Japan; ^8^ Department of Lipid Medical Science Graduate School of Medicine, University of Tokyo Tokyo Japan

**Keywords:** familial Parkinson's disease, α‐Synuclein, genetic screening, protein aggregation, phospholipids

## Abstract

**Background:**

The α‐Synuclein (α‐Syn) V15A variant has been found in two Caucasian families with Parkinson's disease (PD). However, the significance of this missense variant remained unclear.

**Objective:**

We sought to elucidate whether V15A could increase aggregation or change phospholipid affinity.

**Methods:**

A sequencing analysis for the *SNCA* encoding α‐Syn from 875 patients with PD and 324 control subjects was performed. Comparing with known pathogenic missense variants of α‐Syn, A30P, and A53T, we analyzed the effects of V15A on binding to phospholipid membrane, self‐aggregation, and seed‐dependent aggregation in cultured cells.

**Results:**

Genetic screening identified *SNCA* c.44 T>C (p.V15A) from two Japanese PD families. The missense variant V15A was extremely rare in several public databases and predicted as pathogenic using *in silico* tools. The amplification activity of α‐Syn V15A fibrils was stronger than that of wild‐type α‐Syn fibrils.

**Conclusions:**

The discovery of the V15A variant from Japanese families reinforces the possibility that the V15A variant may be a causative variant for developing PD. V15A had a reduced affinity for phospholipids and increased propagation activity compared with wild‐type. © 2022 The Authors. *Movement Disorders* published by Wiley Periodicals LLC on behalf of International Parkinson and Movement Disorder Society

## Graphical abstract

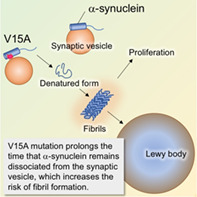

Parkinson's disease (PD) is the second most common neurodegenerative disorder next to Alzheimer's disease.^1^ PD is characterized by bradykinesia, resting tremor, rigidity, and postural instability as motor symptoms, and by constipation, polyuria, and rapid eye movement (REM) behavior disorders as nonmotor symptoms. The pathology shows loss of dopaminergic neurons in the substantia nigra along with Lewy bodies or Lewy neuritis, which are primarily composed of α‐Synuclein (α‐Syn).^1^

α‐Syn is encoded by the *SNCA* gene, which is the first identified pathogenic gene of familial PD with missense variants or copy number variations (ie, duplication^2^ and triplication^3^). α‐Syn is a 140‐amino acid presynaptic protein that comprises three characteristic domains: (1) amphipathic N‐terminal domain, (2) non‐amyloid‐ß component of plaques domain, and (3) acidic C‐terminal domain.^4^ α‐Syn is involved in synaptic vesicle release by binding to the synaptic vesicle membrane through its amphipathic domain, although its physiological function is not fully understood.^5^, ^6^ To date, several pathogenic variants of *SNCA* have been found to be associated with familial PD, including A30P,^7^ A30G,^8^ E46K,^9^ H50Q,^10^ G51D,^11^ A53T,^12^ A53E,^13^ and A53V.^14^ These variants are thought to have the effect of increasing aggregation or changing the affinity for phospholipids. Furthermore, several genome‐wide association studies have demonstrated significant associations between the *SNCA* gene and PD (including sporadic and familial) through large population studies.^15^, ^16^, ^17^

In this study, we identified p.V15A (hereafter V15A), a potential pathogenic variant found in the Caucasian population,^18^ from three affected individuals from two independent Japanese PD families with typical parkinsonism. Our biochemical and cell biological analyses indicated that V15A had a reduced affinity for phospholipids and an increased fibril elongation activity. These properties were of an intermediate degree between the known pathogenic variants and wild‐type (WT). Our findings strongly suggest that V15A is a risk factor for the onset of PD.

## Subjects and Methods

### Study Subjects

This study (H21‐080) was approved by the ethics committee of Juntendo University, Tokyo, Japan, and all subjects provided written informed consent to participate in the genetic research described in this study. All DNA samples were collected from Juntendo PD DNA bank, which were registered from 2016 to 2020. Genomic DNA of 467 patients with PD with familial history, 408 patients with PD without familial history (sporadic PD), and 324 in‐house control subjects were examined. The subjects' demographic data are described in Table 1.

**TABLE 1:** Characteristics of the Subjects Who Underwent Genetic Screening

Subjects	No. of Patients (Male: Female)	Age at Sampling, y ± SD (range)	Age at Onset, y ± SD (range)	Disease Duration, y ± SD (range)
Familial Parkinson's disease	467 (243: 224)	63.1 ± 12.5 (17–94)	56.0 ± 14.0 (12–88)	7.1 ± 7.6 (0–52)
Sporadic Parkinson's disease	408 (232: 176)	50.4 ± 11.4 (14–88)	42.5 ± 10.4 (6–83)	7.9 ± 7.0 (0–37)
In‐house control	324 (111: 213)	62.3 ± 16.0 (20–98)		

PD was clinically diagnosed based on standard clinical diagnostic criteria.^19^, ^20^ We obtained the clinical information from each attending doctor via questionnaire or medical records. The Unified Parkinson's Disease Rating Scale (UPDRS) was used to evaluate symptoms.

### Genetic Studies

Genomic DNA was extracted from peripheral blood using a standard protocol. Targeted panel resequencing (IAD103177_182) via Ion Torrent system (Thermo Fisher Scientific, Waltham, MA, USA) was used to screen *SNCA* variants in patients with PD and control subjects, as reported previously.^21^ Pathogenic or likely pathogenic variants detected by this panel were confirmed by Sanger sequencing. Copy number variations in *SNCA* were also analyzed using multiplex ligation‐dependent probe amplification (MLPA) methods with SALSA MLPA Probemix P051/P052 (MRC‐Holland, Amsterdam, the Netherlands).

Whole‐exome sequencing and its analysis were conducted by the standard method using the samples of four members in family A (II‐10, III‐10, III‐11, III‐12) and one member in family B (III‐1). Sequencing details are described in the Supporting Information.

Haplotype analysis was performed to determine the likelihood of a common founder among four patients harboring V15A. These details are described in Supporting Information Table S1.

### In Silico Analysis

**FIG 1:**
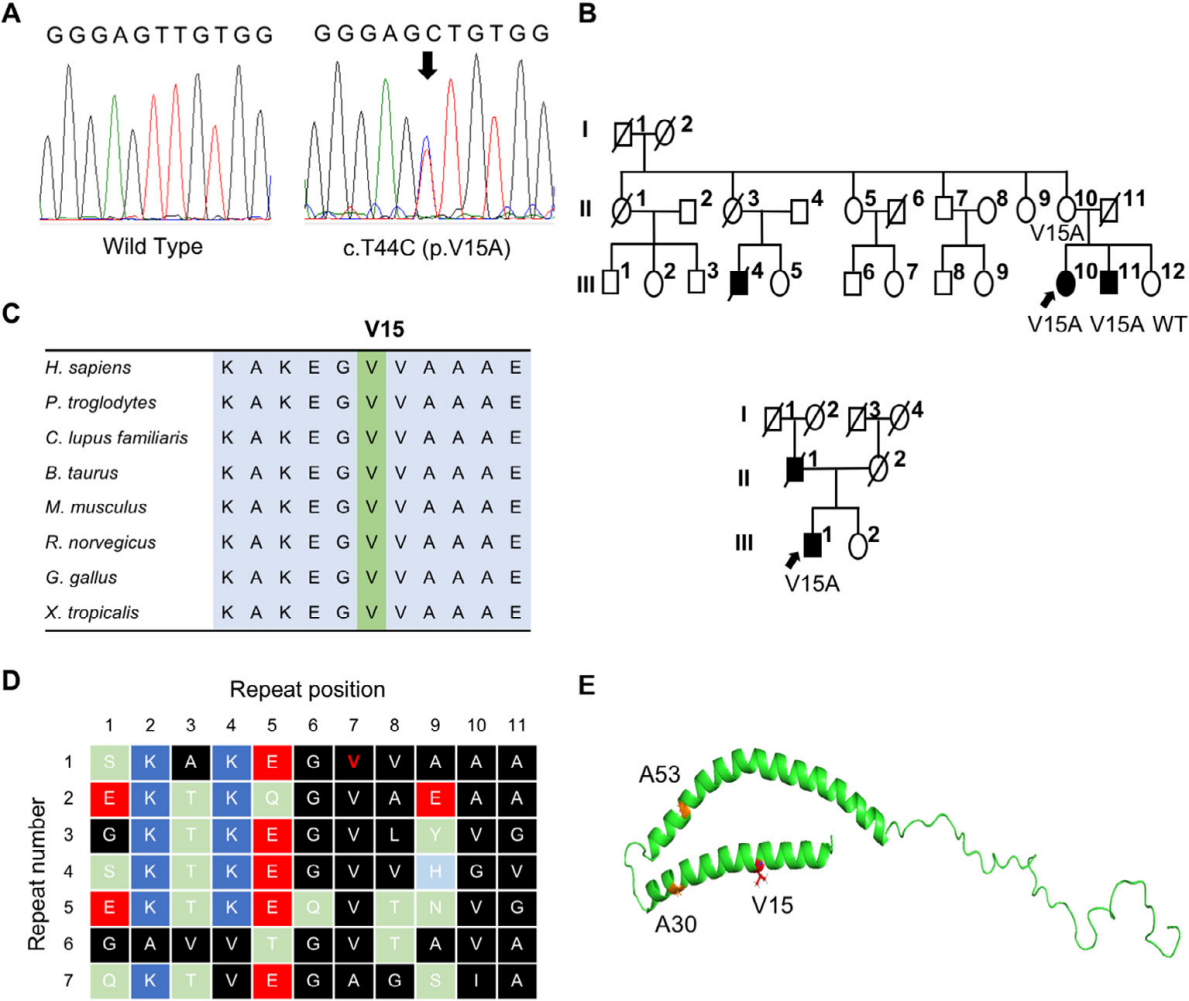
V15A (c.44 T>C) variant in the *SNCA* gene. (**A**) Electropherogram of Sanger sequence detects c.44 T>C (arrow) in the *SNCA* gene. (**B**) Family pedigree of families with *SNCA* V15A: upper, family A; lower, family B. Arrows, probands; squares, men; circles, women; oblique lines, deceased; black squares and circles, clinically diagnosed with PD. (**C**) Alignment of α‐Syn amino acid sequence containing V15 among species. (**D**) Characteristic KTKEGV repeats of α‐Syn. Blue, basic; red, acidic; light green, polar uncharged; black, nonpolar residues. Affected V15 is indicated in red. (**E**) The three‐dimensional structures of α‐Syn, in which affected amino acids are indicated. [Color figure can be viewed at wileyonlinelibrary.com]

Frequency and effect of V15A were investigated using several public databases and prediction tools written in the Supporting Information.^22^, ^23^, ^24^, ^25^, ^26^ α‐Syn orthologues were aligned using the National Center for Biotechnology Information's HomoloGene database (https://www.ncbi.nlm.nih.gov/homologene). Structures of human α‐Syn bound to micelle in Fig. 1E and Supporting Information Fig. S3 were depicted based on Protein Data Bank (PDB): 1XQ8 using PyMOL (The PyMOL Molecular Graphics System, Version 2.0.7, Schrödinger, NY, USA).^27^

### Preparation for α‐Syn Plasmids

V15A, A30P, and A53T were introduced in the bacterial expression constructs pRK172‐human α‐Syn^28^ and pGEX‐6P‐1‐human α‐Syn, and in the mammalian expression construct pcDNA3‐human α‐Syn; this was completed via site‐directed mutagenesis with PrimeSTAR Max (Takara Bio, Kusatsu, Japan). The primers for mutagenesis are described in Supporting Information Table S2.

### Recombinant α‐Syn Purification

For the liposome binding assay, human α‐Syn was cloned into EcoRI and XhoI sites of pGEX‐6P‐1. Production and purification of glutathione *S*‐transferase (GST)‐fusion α‐Syn was performed according to the manufacturer's protocol (Cytiva, Tokyo, Japan).

For fibril assembly, self‐coagulation, and seed preparation, recombinant α‐Syn was purified from bacteria as described previously.^29^ These details are described in the Supporting Information.

### Preparation of Fluorescence‐Labeled Liposomes

Liposomes were prepared from 1,2‐dioleoyl‐sn‐glycero‐3‐phosphocholine/1,2‐dioleoyl‐sn‐glycero‐3‐phospho‐l‐serine (7:3) and 3,30‐dioctadecyloxacarbocyanine perchlorate (0.5% w/v). Preparation of fluorescence‐labeled liposomes was conducted as described previously.^30^, ^31^ Preparation details are described in the Supporting Information.

### Liposome Binding Assay

The liposome binding assay was completed as per previous reports.^30^, ^32^, ^33^ GST‐tagged α‐Syn (37.5 pmol) bound to 30 µl glutathione sepharose beads (Cytiva) in 100 µl HBSE (20 mM 2‐[4‐(2‐Hydroxyethyl)‐1‐piperazinyl]ethanesulfonic acid [pH 7.3], 100 mM NaCl, and 1 mM ethylenediaminetetraacetic acid) was incubated with 75 μM liposomes at 37C for 7.5 minutes. After washing the glutathione beads with 1000 μL HBSE at 4C three times, the beads were incubated with 100 μL HBSE containing 0.2% Triton X‐100 to release the liposomes. The resultant supernatant was transferred to a 96‐well microplate, and liposome fluorescence was measured at 460 nm excitation/538 nm emission using SpectraMax iD3 (Molecular Devices, San Jose, CA, USA). GST alone was used as a control. The binding capacity was calculated using the following formula:

Binding ratio = (GST‐tagged α‐Syn X − GST alone)/(GST‐tagged α‐Syn WT − GST alone).

### Electron Microscopy Analysis of α‐Syn Fibrils

Filament assembly was conducted according to a previous report.^29^ Details are described in the Supporting Information.

### Real‐Time Quaking‐Induced Conversion

We measure the self‐coagulation ability of α‐Syn by real‐time quaking‐induced conversion (RT‐QUIC)^34^; details are described in the Supporting Information. The fluorescence intensity of thioflavin T was recorded to evaluate the self‐coagulation ability. The thioflavin T fluorescence threshold was defined as the average fluorescence intensity of all samples at time 0 + 3 standard deviations (SDs), and the maximum fluorescence intensity was set to 260,000. The positive time in Fig. 3B was calculated as the time when the fluorescence intensity reached the threshold.

### α‐Syn Seeding Assay

α‐Syn seed preparation and introduction of the seeds into SH‐SY5Y cells were performed as described previously.^29^ pcDNA3‐α‐Syn plasmid was introduced into SH‐SY5Y cells using the X‐tremeGENE 9 DNA transfection reagent (Roche, Basel, Switzerland). After incubation for 3 days, α‐Syn was sequentially extracted using four different buffers.^29^ Sarkosyl‐soluble and ‐insoluble fractions were subjected to 15% SDS‐PAGE gel/western blotting. Sarkosyl‐soluble and ‐insoluble α‐Syn were detected using anti‐α‐Syn (ab138501; Abcam, Cambridge, UK) and anti‐phospho‐Ser129 α‐Syn antibody (ab51253; Abcam), respectively. Band intensity of phospho‐Ser129 α‐Syn was measured using ImageJ (National Institutes of Health, Bethesda, MD, USA).^35^

### Data Analysis and Statistics

Statistical analysis was performed using JMP 16 (SAS Institute Inc., Cary, NC, USA). Comparison of WT with variants was analyzed by Dunnett's test. Comparison among all four types of α‐Syn was conducted by Tukey–Kramer's test.

## Results

### Identification of V15A in *SNCA*

Our genetic screening identified two probands from families A and B, harboring c.44 T>C, p.V15A in *SNCA*, among 467 familial PD, 408 sporadic PD, and 324 control subjects (Fig. 1A). There were no pathogenic variants among patients with sporadic PD and control subjects.

We subsequently conducted a segregation study for the affected family members. The V15A variant was identified in three members of family A (II‐10, III‐10, III‐11), but not in an unaffected sibling (III‐12) (upper in Fig. 1B). In family B, a DNA sample was available only for the proband (lower in Fig. 1B). The allele frequency of the V15A variant was 0.00000658 in gnomAD. There was no record of V15A in the Japanese population database (GEM‐J WGA, 14KJPN jMorp) (Supporting Information Table S3). The variant was predicted as deleterious using three different prediction tools (Supporting Information Table S3). The residue is highly conserved across many species (Fig. 1C). No copy number variations in *SNCA* were found by the MLPA method.

Whole‐exome sequencing was also performed on members of family A (II‐10, III‐10, III‐11, III‐12) and family B (III‐1). We found no common pathogenic variant related to PD. Haplotype analysis showed that carriers of V15A in families A and B shared a 1.2‐Mb region around the variant, suggesting the presence of a common founder in families A and B (Supporting Information Table S4). However, it should be noted that determining the exact haplotype phase of family B was difficult because only the proband's DNA was available.

### Clinical Findings of Subjects with V15A

#### Family A: Subject III‐10

In family A, three members (III‐4, III‐10, III‐11) were affected by PD (upper in Fig. 1B). III‐10 presented right‐hand resting tremor at the age of 42 years. She was diagnosed with PD at age 44, showing obvious improvement by levodopa administration. III‐10 was admitted to the hospital for drug adjustment against wearing off and levodopa‐induced dyskinesia at age 52. At age 57, she showed mild cognitive impairment (Japanese version of the Montreal Cognitive Assessment [MoCA‐J], 24/30 points; Mini‐Mental State Examination [MMSE], 27/30 points). By this point, degeneration of subject III‐10's motor skills had progressed to the point that she needed to use a wheelchair for daily activities. Regarding autonomic dysfunction, subject III‐10 presented with orthostatic hypotension, and her index of the Modified Hoehn & Yahr (H&Y) stage was 5. III‐10 was dead at age 62.

**FIG 2:**
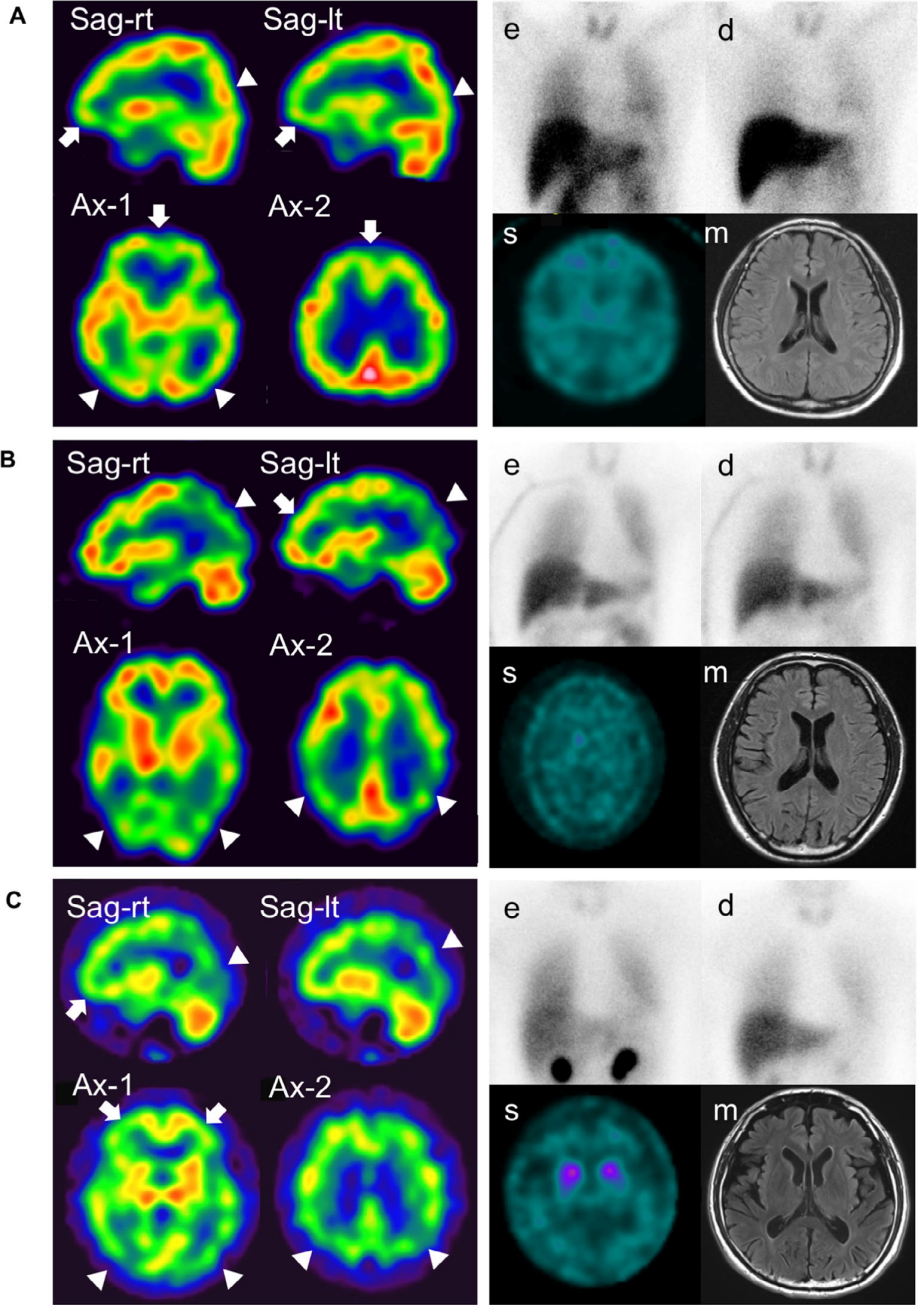
Clinical images of carriers with *SNCA* p.V15A. (**A**) Family A subject III‐10. (**B**) Family A subject III‐11. (**C**) Family B subject III‐1. Left: single‐photon emission computed tomography (SPECT) with N‐isopropyl‐p‐[^123^I]‐iodoamphetamine (IMP‐SPECT); upper right: ^123^iodine‐metaiodobenzylguanidine myocardial scintigraphy at early (e) and delay (d) phases; lower right: SPECT imaging with ^123^I‐ioflupane (s) and brain magnetic resonance imaging of fluid‐attenuated inversion recovery (m). Arrows and arrowheads indicate decreased perfusion in the frontal lobe and in the occipital lobe, respectively. Images of Ax‐1 and Ax‐2 contain the basal ganglia and the body of the lateral ventricle, respectively. Ax, axial; lt, left; rt, right; Sag, sagittal. [Color figure can be viewed at wileyonlinelibrary.com]

Brain magnetic resonance imaging (MRI) showed no abnormalities (Fig. 2A). ^123^Iodine‐metaiodobenzylguanidine (MIBG) myocardial scintigraphy indicated a decreased heart‐to‐mediastinum (H/M) ratio (early 2.10, delay 1.79) (Fig. 2A). Dopamine transporter single‐photon emission computed tomography with ^123^I‐ioflupane (DAT‐SPECT) showed a decrease in both basal ganglia (Fig. 2A). Brain SPECT with N‐isopropyl‐p‐[^123^I]‐iodoamphetamine (IMP‐SPECT) showed hypoperfusion of blood flow in the frontal and posterior lobes (Fig. 2A).

#### Family A: Subject III‐11

Subject III‐11 was the younger brother of subject III‐10 (upper in Fig. 1B). He presented with right‐dominant hand tremor and first visited the neurology clinic at age 50 years. His symptoms improved considerably with levodopa. Subject III‐11 was diagnosed with PD that was well controlled by levodopa. By 5 years from onset, subject III‐11 had gradually experienced wearing off and urinary incontinence. He started taking catechol‐*o*‐methyl‐transferase inhibitor at age 55 years. At age 58, subject III‐11's UPDRS Part III scores were 18/108 and 28/108 points at *on* and *off* phases, respectively. His MoCA‐J score was 24/30, suggesting cognitive decline. Subject III‐11's H&Y stage was 3 at the study conclusion at age 60. III‐11's brain MRI was normal (Fig. 2B). MIBG myocardial scintigraphy indicated a decreased H/M ratio (early 1.85, delay 1.39), and DAT‐SPECT showed a decrease in bilateral basal ganglia (Fig. 2B). IMP‐SPECT indicated decreased blood flow in the posterior lobe (Fig. 2B).

#### Family A: Other Subjects

Subject III‐4 (upper in Fig. 1B) was also diagnosed with PD and died at age 54. Detailed information for this subject was not available.

Subject II‐10 (upper in Fig. 1B), who was the mother of subjects III‐10 and III‐11, also harbors V15A. However, she did not present with any parkinsonism or cognitive decline until age 85. Her DAT‐SPECT imaging and brain MRI did not show any abnormality.

Noncarrier III‐12 (upper in Fig. 1B), who is the younger sister of subjects III‐10 and III‐11, did not present any symptoms by the study's conclusion at age 49.

#### Family B: Subjects II‐1

There were two patients with PD in family B (lower in Fig. 1B). Subject III‐1 showed cognitive decline and symptoms related to REM behavior disorders at age 55 years. At age 59, he developed gait disturbance and subsequently visited the neurology clinic with right‐dominant akinesia and cognitive decline (MMSE, 23 points; MoCA‐J, 16/30 points). At 60 years old, subject III‐1 presented with vivid visual hallucinations and was clinically diagnosed as having dementia with Lewy bodies (DLB).^36^ At age 62, his indices of neurological tests were 2 in H&Y stage at the *on* phase, 22/108 of UPDRS Part III at the *on* phase, and 22/30 of MMSE. Brain MRI showed normal findings, while DAT‐SPECT showed a decrease in bilateral basal ganglia (Fig. 2C). The H/M ratio of MIBG myocardial scintigraphy decreased (early 1.97, delay 1.59), and IMP‐SPECT showed hypoperfusion in the frontal and the posterior regions (Fig. 2C).

Subject III‐1's father (II‐1) was also diagnosed with PD and died at age 68 years, although additional details about this case were not available.

#### Families A and B: Additional Details

PD carrier information for the two families is summarized in Table 2.

**TABLE 2:** Clinical Features of Subjects with *SNCA* V15A

	Family A		Family B
	III‐10	III‐11	III‐1
AAO	42	50	59
AAE	58	58	62
Sex	F	M	M
Initial symptom	Tremor	Tremor	Gait disturbance
Hoehn & Yahr	5	3	2
Resting tremor	+	+	−
Bradykinesia	+	+	+
Rigidity	+	+	+
Postural instability	+	+	−
Gait disturbance	+	+	+
Hesitation	+	+	−
Response to levodopa	+	+	+
Wearing off	+	+	−
*On*/*Off*	+	+	−
Levodopa‐induced dyskinesia	+	−	−
Asymmetry of onset symptom	+	+	−
Dystonia at onset	−	−	−
Dystonia showing response to levodopa	−	−	−
Hyperreflexia	−	−	−
Constipation	−	+	−
Urinary disturbance	−	+	−
Orthostatic hypotension	+	−	−
Sleep benefit	−	−	−
Gaze palsy	−	−	−
Cerebellar ataxia	−	−	−
Hallucination	−	−	+
Delusion	−	−	−
Depression	+	−	−
Cognitive decline	+	+	+
HDS‐R	28	18	23
MMSE	27	23	19
MoCA‐J	24	24	16
Mental retardation	−	−	−
Nightmare	−	−	+
REM sleep behavior disorder	−	−	+
Sudden sleep	−	−	−
Restless legs syndrome	+	−	−
Olfactory dysfunction	−	−	−
Pain/Sensory disturbance	−	−	−
MRI	Normal	Normal	Normal
IMP‐SPECT	Decrease in frontal and occipital lobe	Decrease in occipital lobe	Decrease in frontal and occipital lobe
DAT‐SPECT	Decrease	Decrease	Decrease
MIBG	Decrease intake	Decrease intake	Decrease intake
Diagnosis	PD	PD	DLB
UPDRS Part I	2	1	NA
UPDRS Part II	37	13	NA
UPDRS Part III	34	28	22
UPDRS Part IV	10	2	NA

AAO, age at onset; AAE, age at examination; F, female; M, male; HDS‐R, Hasegewa Dementia Scale–Revised; MMSE, Mini‐Mental State Examination; MoCA‐J, Japanese version of the Montreal Cognitive Assessment; REM, rapid eye movement; MRI, magnetic resonance imaging; IMP‐SPECT, single‐photon emission computed tomography with N‐isopropyl‐p‐[^123^I]‐iodoamphetamine; DAT‐SPECT, dopamine transporter single‐photon emission computed tomography with ^123^I‐ioflupane; MIBG, ^123^iodine‐ metaiodobenzylguanidine myocardial scintigraphy; PD, Parkinson's disease; DLB, dementia with Lewy bodies; UPDRS, Unified Parkinson's Disease Rating Scale; NA, not available.

### α‐Syn V15A Shows a Decreased Affinity to Phospholipids

V15A has so far been reported in two families with PD^18^, ^37^ and is suspected to be pathogenic according to its rare frequency and pathogenic prediction by *in silico* tools (Supporting Information Table S3). However, its effect on the α‐Syn protein remains unknown.

**FIG 3:**
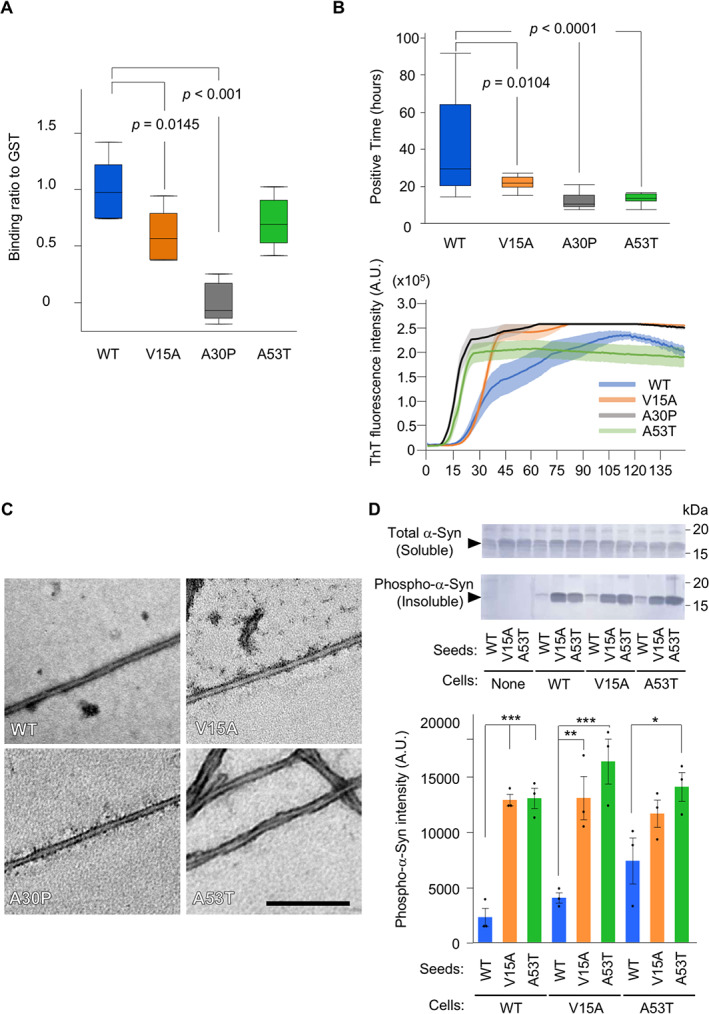
Pathogenic properties of α‐Synuclein (α‐Syn) V15A. (**A**) Liposome binding efficiency of α‐Syn variants. Box plots indicate the 25th to 75th percentiles of the binding ratio (n = 6). Horizontal lines in the boxes indicate the 50th percentile, and whiskers represent the maximum and minimum values. (**B**) RT‐QUIC to evaluate the self‐coagulation ability of α‐Syn. The graph (n = 15 in each) represents the time to reach a given threshold fluorescence intensity. (**C**) Morphology of α‐Syn fibrils evaluated by electron microscopy. Scale bar: 200 nm. (**D**) α‐Syn in 1% sarkosyl‐soluble fraction (soluble) and ‐insoluble fraction (insoluble). Graph represents the band intensity of phospho‐S129 α‐Syn (n = 3). *P < 0.05; **P < 0.01; ***P < 0.001, by Tukey–Kramer's test. A.U., arbitrary units. [Color figure can be viewed at wileyonlinelibrary.com]

We next analyzed the biochemical properties of V15A α‐Syn. When α‐Syn binds to phospholipids, its N‐terminal region forms an amphipathic helix, stabilizing its structure.^6^ This lipid‐binding property assists in the assembly of soluble N‐ethylmaleimide‐sensitive factor (NSF) attachment protein receptor proteins involved in synaptic vesicle exocytosis.^38^ Conversely, once dissociated from phospholipids, α‐Syn does not take a constant form,^6^ which is thought to be an unstable state that leads to a conversion of the ß sheet structure.^31^ We thus evaluated the effect of V15A on the phospholipid‐binding property using 1,2‐dioleoyl‐sn‐glycero‐3‐phosphocholine/1,2‐dioleoyl‐sn‐glycero‐3‐phospho‐l‐serine liposomes. As reported previously,^30^ the affinity for phospholipids was greatly reduced in A30P compared with WT and A53T. Under the same conditions, V15A showed a mild decrease in the binding (*P* = 0.0139; Fig. 3A).

### α‐Syn V15A Has an Increased Aggregation Property Compared with WT

Aggregation and prion‐like propagation of α‐Syn is thought to be a major cause of PD.^39^, ^40^ We evaluated the aggregation susceptibility of V15A by RT‐QUIC, which monitors the assembly of α‐Syn fibrils quantitatively (Fig. 3B). The fluorescence intensity of A53T α‐Syn reached a threshold at an early time, indicating stable fibril amplification, as previously reported.^30^ Under our buffer conditions, V15A α‐Syn showed an increased aggregation activity compared with WT but was weaker than A30P and A53T (Fig. 3B and Supporting Information Fig. S1). The seeding activity of V15A α‐Syn fibrils using WT α‐Syn as a substrate was also higher than that of WT fibrils (Supporting Information Fig. S2). WT, V15A, and A30P α‐Syn fibrils had a rod‐like appearance of approximately 10 nm in width (Fig. 3C), while A53T α‐Syn fibrils had a characteristic twisted appearance (Fig. 3C). These results indicate that V15A α‐Syn forms fibrils with the same structure as WT α‐Syn.^41^

### α‐Syn V15A Has an Enhanced Seeding Activity

α‐Syn fibrils have prion‐like propagation activity and disrupt neural circuits. This activity is thought to be associated with PD progression and has been analyzed in cellular and animal models.^29^, ^42^, ^43^, ^44^

We compared the seeding activity of three kinds of α‐Syn (WT, V15A, and A30P) in SH‐SY5Y cells, where α‐Syn WT, V15A, or A30P was overexpressed (Fig. 3D). V15A seeds induced stronger aggregation in SH‐SY5Y cells expressing α‐Syn WT or V15A compared with WT seeds; the result was almost the same as that of the A53T seeds (Fig. 3D). A53T exhibited the strongest property in terms of cross‐seeding activity in cells. These results suggest that once V15A fibrils are generated, fibril elongation may easily occur even in the presence of α‐Syn WT.

## Discussion

Our study provides genetic and biochemical evidence of the α‐Syn V15A variant as a cause of familial PD. We identified V15A among three patients in two Japanese families. They shared a common haplotype, which may suggest a common founder between the patients. The variant was quite rare in public databases (allele frequency < 0.00001) and was not identified in in‐house controls. V15 residue is highly conserved among species. Biochemical studies showed pathogenic properties of V15A, which included decreased affinity to phospholipid membrane and accelerated seed‐dependent aggregation in cells. These results support our conclusion that V15A is a pathogenic variant.

Patients with V15A in our study develop typical PD at the age of 42–59 years (mean ± SD, 50 ± 8.50 years). All three subjects in our study showed good response to levodopa and often presented with cognitive decline. Motor fluctuation and autonomic dysfunction were found in two of the subjects. MIBG myocardial scintigraphy, one of the biomarkers for the presence of Lewy bodies, showed a decreased intake in all three subjects (Fig. 2). This result is consistent with the pathology reports of the *SNCA* missense variant presenting with abundant Lewy bodies.^45^ Low uptake of IMP‐SPECT in the occipital lobe, which is a supportive biomarker of DLB, was found in all three subjects.^36^, ^46^ These findings suggest DLB might be a common phenotype of V15A. It is notable that Subject II‐10 in family A did not present with any symptoms until the age of 85 years. The asymptomatic carrier suggests incomplete penetrance of V15A.

There are two reports in the literature describing patients with PD with V15A (Supporting Information Table S5).^37^, ^47^ The first article reported a patient with familial PD harboring V15A, whose age at onset (AAO) was 59 years.^37^ He presented with right‐dominant parkinsonism; partial response to levodopa; cognitive decline; and psychological symptoms, including abulia, agitation, visual hallucination, hyposmia, and REM sleep behavior disorder. Two of his three siblings also developed PD and dementia; however, they were not examined via genetic testing. Another older sibling was an unaffected carrier.

In the second report, three of four siblings harboring V15A developed PD in an Italian family.^47^ The proband developed PD at age 47 years, presenting with an excellent response to levodopa and motor dyskinesia, accompanied by cognitive decline starting 6 years before onset. Two of three of her siblings also developed rigid‐akinesia parkinsonism at age 50 years, without cognitive decline. One of her siblings, also harboring V15A, presented with depression and anxiety without motor symptoms of PD. These patients' parents, who died at the ages of 60 and 40 years, were not diagnosed with PD. V15A was genetically confirmed in all siblings of the proband. The AAO of the Italian family was 49.0 ± 1.73 years (mean ± SD), which was similar to that of the affected family members in our study.

*SNCA* pathogenic missense mutation variants have high age‐dependent disease penetrance, and meta‐analysis has assumed 100% penetrance by the age of 80 years.^48^ Asymptomatic carriers were found in both of the earlier Italian studies. Considering our cases and the Italian reports, the penetrance of V15A might be not as high as with previously reported *SNCA* missense mutations.

The V15D variant was found from one patient with sporadic PD from China, although its clinical phenotype was not reported.^49^ Thus, this study is the first report of V15A in Asian familial PD. To summarize the symptoms, PD with V15A is characterized by early onset (40s to 50s), cognitive impairment, dyskinesia, visual hallucination, and good response to levodopa. There are four families in our study and previous reports, comprising seven patients with PD.

Comparing the clinical symptoms of patients with V15A with other missense variants of *SNCA*, the mean AAO of patients with PD with V15A seems to be later than A53T, earlier than A30P, and similar to that of E46K (Supporting Information Table S6).^50^, ^51^ Time to death from motor onset was 20 years in one patient with V15A (subject III‐10 in family A), and the remaining patients (subject III‐11 in family A and subject III‐1 in family B) are currently alive at 11 and 4 years from onset, respectively.

The progression of patients with V15A might be relatively slow in patients with *SNCA* missense mutation.^51^ The frequency of cognitive decline in V15A patients (all three patients in our study) appears to be higher than with other missense mutations. This finding indicates that cognitive impairment could be a characteristic symptom of V15A. The subjects in our study and in the Italian family responded well to levodopa, which is similar to A30P cases, of which symptoms are similar to those of sporadic late‐onset PD.^47^ These findings suggest that the severity in clinical symptoms of patients with V15A might be intermediate in patients with known *SNCA* pathogenic mutations, although there is clinical variability in *SNCA* missense mutations.^52^ It is important to study additional cases to clarify the clinical phenotype of V15A.

How the binding properties of α‐Syn to phospholipids affect α‐Syn aggregation is a matter of debate. For example, A30P loses its binding properties to phospholipids, while E46K binds strongly to phospholipids.^9^, ^30^, ^53^ These observations suggest that alteration of the phospholipid‐binding property might be an essential factor for α‐Syn aggregation. When disassociated from phospholipids, α‐Syn is in an intrinsically disordered state and is thermodynamically unstable.^54^ This possibility is supported by the fact that there is self‐aggregation of α‐Syn in the absence of phospholipids *in vitro*. In this state, changes in the external environment would increase the likelihood of cross‐β sheet conformation.

*PARK14* is a familial PD that exhibits prominent Lewy body deposition.^31^ Variants of *PLA2G6/phospholipase A*_*2*_ *VI*, the gene responsible for *PARK14*, lead to shortening of the acyl groups of phospholipids, weakening the binding of α‐Syn to phospholipids.^31^ This observation suggests that dissociation from phospholipids is a risk for α‐Syn aggregation. A longer dissociation state of A30P from phospholipids would be a risk for aggregation. E46K, which binds strongly to phospholipids, forms a stable amphipathic helix when bound to phospholipids.^30^ However, once dissociated from phospholipids, it would show strong aggregation properties.^30^ Alternatively, partial binding of α‐Syn on the phospholipid membranes via its N‐terminal 3–25 residues may destabilize the structure of the non‐amyloid‐ß component region, leading to a cross‐β structure.^55^

Our phospholipid binding assay suggested a weaker affinity of V15A α‐Syn to the phospholipid membrane. The N‐terminal region of α‐Syn is characterized by seven 11‐residue repeats, with a conserved KTKGEV motif (Fig. 1D).^56^ The repeats form an alpha‐helical structure that enables α‐Syn to bind to acidic phospholipids stably.^57^, ^58^ V15, as well as A30P, locates in the hydrophobic side of the first amphipathic helix, which is more important for phospholipid binding than the second amphipathic helix (Fig. 1E).^55^, ^59^ Substitution of V15 with A may cause a slight decrease in hydrophobicity, which may lead to dissociation from the membrane (Supporting Information Fig. S3). Alternatively, V15A might cause a helix‐breaking effect, such as that of A30P, although its effect is expected to be small.^60^

The effects of V15A on fibril amplification were evaluated by RT‐QUIC under a physiological salt condition, assuming a state that α‐Syn is released from phospholipids and self‐aggregates. The V15A aggregation property was stronger than that of WT, but it was weaker than that of A53T and A30P. The seed‐dependent aggregation assay in cultured cells suggests that the pathogenic property was stronger in the order of A53T, V15A, and WT. These results appear to correspond to clinical severity of PD with *SNCA* variants, including V15A.

There are two major limitations in this study. The first issue is that the sample size of V15A carriers is small. Further screenings among various ethnic groups would be required to determine the character of V15A with respect to pathogenicity. The second issue is the method of *in vitro* aggregation assay (modified RT‐QUIC) that we used. The salt concentration is critical for the aggregation of α‐Syn and may also affect the aggregation property of missense variants.^61^ Although we used physiological salt concentrations, further validation is needed to determine whether the results accurately reflect *in vivo* conditions.

In conclusion, we discovered the α‐Syn V15A variant in two Japanese families with PD. V15A weakened the α‐Syn binding to phospholipid membranes and promoted α‐Syn aggregation, showing intermediate properties between WT and known pathogenic mutants A30P and A53T. Our clinical and biochemical data underlie the importance of the first amphipathic helix of α‐Syn and can help researchers and clinicians understand how missense variants in α‐Syn are involved in the pathogenesis of PD.

## Author Roles

(1) Research Project: A. Conception, B. Organization, C. Execution.

(2) Manuscript: A. Writing of the first draft, B. Review and Critique.

(3) Other: A. Data analysis, B. Clinical data collection.

K.D.: 1A, 1C, 2A, 3A, 3B.

S.S.: 3A, 3C, 3D.

K.S.‐F.: 3A.

J.O.: 3A.

H.Y.: 1A, 1B.

A.O.: 3B.

T. Hatano: 3B.

Y.M.: 2A‐C.

T. Hirunagi: 1A, 2B.

M.K.: 1A, 2B.

H.S.: 3A.

M.F.: 1A, 1B.

K.N: 1A, 1B.

N.H.: 1A, 1B, 2A‐C.

Y.I.: 1A, 1B, 2A, 3A.

## Financial Disclosures

Nothing to report.

## Supporting information


**Appendix S1.** Supporting information

## Data Availability

The data that support the findings of this study are available from the corresponding author, Yuzuru Imai, on reasonable request.
