# Supplementary material for: α‐Synuclein V15A Variant in Familial Parkinson's Disease Exhibits a Weaker Lipid‐Binding Property
Source: Mov Disord. 2022 Jul 27;37(10):2075–85. doi: 10.1002/mds.29162 (PMC9796804; doi:10.1002/mds.29162)
Supplement: Supplementary file 1 — Appendix S1. Supporting information [file MDS-37-2075-s001.pdf]

Supporting Information

Methods

Whole-exome Sequencing

Sample preparation for whole exome sequencing was performed using the SureSelect Human All Exon V6 Kit (Agilent Technologies, Santa Clara, CA, USA). Paired-end sequencing with 150 bp per read of four subjects（II-10, III-10, III-11, III-12) in Family A and one affected subject with V15A in Family B (III-1) was performed using NovaSeq6000 (Illumina, San Diego, CA, USA). Read alignment to human genome (UCSC GRch38/hg38) with Burrows-Wheeler Aligner version 0.7.17-r1188 was referred. Single nucleotide variants were detected in each patient using SAM tools version 1.10. Variant calling, indel realignment, and base quality score recalibration were performed with GATK v.4.1.3.0. Variants identified by whole-exome sequencing were filtered according to the following criteria: base quality score, location in exons or splice sites; the allele frequency in public databases (gnomAD) smaller than 0.001.

Haplotype Analysis

A panel of 16 microsatellites spanning a distance of 1.9 Mb regions across V15A was used (Supporting Information Table S1). Microsatellites were amplified by PCR using fluorescently labeled forward primers, run on an ABI PRISM 3130 Genetic Analyzer, and analyzed using the GeneMapper 4.0 software (Applied Biosystems, Foster City, CA, USA).

In Silico Analysis

V15A frequency was investigated using the Genome Aggregation Database (gnomAD),[1] GEM Japan Whole Genome Aggregation (GEM-J WGA) Panel (Available from: https://togovar.biosciencedbc.jp/doc/datasets/gem_j_wga.) and 14KJPN from the Japanese Multi Omics Reference Panel (14KJPN jMorp).[2] The effect of the V15A missense variant on α-Syn structure was estimated using polyphen2,[3] sorting intolerant from tolerant,[4] and Mutation Taster for the prediction analysis of amino-acid changes.[5]

Recombinant α-Syn Purification

For the liposome binding assay, human α-Syn was cloned into the EcoRI and XhoI sites of pGEX-6P-1. Production and purification of N-terminal glutathione S-transferase (GST)-fusion α-Syn was performed according to the manufacturer's protocol (Cytiva, Tokyo, Japan). The N-terminal GST tag does not affect the binding of α-Syn to lipids.[6-8] For fibril assembly, self-coagulation, and seed preparation, recombinant α-Syn was purified from bacteria as described previously.[9] *E. coli* BL21 harboring pRK172-human α-Syn incubated on an

LB agar plate overnight was inoculated in 500 ml LB medium. Bacteria were cultured at 200 rpm at 37°C for 3.5 hours and α-Syn was induced with 100 mM isopropyl β-D-thiogalactopyranoside (IPTG) for 7.5 hours. Bacterial pellets were suspended and sonicated in α-Syn purification buffer (50 mM Tris HCl, pH 7.4, 1 mM EGTA, 1 mM dithiothreitol). After centrifugation at 6000 *g* for 10 minutes, the supernatant containing 1% 2-mercaptoethanol was incubated in boiling water for 5 minutes. The resultant solution was centrifugated at 20000 *g* for 15 minutes. The supernatant was loaded onto Q Sepharose column (Cytiva) and eluted with α-Syn purification buffer containing 350 mM NaCl. The elution was salted out with ammonium sulfate and dialyzed against 30 mM 3-morpholinopropanesulfonic acid (MOPS), pH 7.2.

Preparation of Fluorescence-labeled Liposomes

1,2-Dioleoyl-sn-glycero-3-phosphocholine (DOPC) and 1,2-dioleoyl-sn-glycero-3-phospho-L-serine (DOPS) were purchased from Avanti Polar Lipids (Alabaster, AL, USA). 3,30-Dioctadecyloxacarbocyanine perchlorate (DiO) was purchased from Merck (Darmstadt, Germany). DOPC/DOPS (7:3) and DiO (0.5% w/v) in chloroform-methanol were dried using a sample concentrator WD-12 (Hangzhou Allsheng Instruments, Hangzhou, China) and suspended in HBSE (20 mM HEPES, pH7.3, 100 mM NaCl, and 1 mM EDTA). After 5 freeze-thaw cycles of the suspension, liposomes were prepared by passing through a mini-extruder equipped with a 50-nm filter (Avanti Polar Lipids) 21 times.

Real-time Quaking-induced Conversion (RT-QUIC)

The self-coagulation ability of α-Syn was measured by RT-QUIC.[10] For Fig. 3B, recombinant α-Syn cleaned by ultracentrifugation at 100,000 *g* for 20 minutes was resolved in a reaction buffer [40 mM sodium phosphate buffer, pH8.0, 150 mM NaCl, 0.1% NaN_3, 10 mM thioflavin T (ThT)] to a final concentration of 0.75 mg/ml. Zirconium/silica beads (16 mg/well, 0.5 mm in diameter; BioSpec Products, Bartlesville, OK, USA) and 100 µl α-Syn solution were dispensed in a 96-well optical bottom plate (#265301, Thermo Fisher Scientific). Fluorescence was measured using a FluoSTAR OMEGA plate reader (BMG Labtech, Ortenberg, Germany) with the settings of 42°C with intermittent double orbital shaking at 400 rpm for one minute, followed by one minute rest. Fluorescence intensity of ThT was recorded every 45 minutes using 450-nm excitation/480 nm emission filters. The ThT fluorescence threshold was defined as the average fluorescence intensity of all samples at time 0 + 3 standard deviations (SD), and the maximum fluorescence intensity was set to 260000. The bottom graph in Fig. 3B represents the mean ± SEM. The positive time in Fig. 3B was calculated as the time when the fluorescence intensity reached the threshold.

α-Syn WT or V15A seeds were prepared from recombinant α-Syn protein produced in bacteria as described previously (Supporting Information Fig. S2).[9] Then, α-Syn seeds diluted from 150 ng to 1.5 µg were resolved in 100 µl reaction buffer containing 100 mM phosphate buffer at pH 8.2, 10 µM ThT, 100 µg/ml recombinant α-Syn WT, and 37 mg zirconium/silica beads. Fluorescence

was measured using a FluoSTAR OMEGA plate reader with the following settings: 30°C with intermittent double orbital shaking at 200 rpm for 1 minute, followed by 14 minutes rest. Fluorescence intensity of ThT was recorded every 15 minutes.

Electron Microscopy Analysis of α-Syn Fibrils

Recombinant α-Syn (8 mg/ml) in 30 mM MOPS, pH 7.2, containing 0.01 % NaN_3 was shaken at 200 rpm at 37°C for 7 days using a Bio Shaker BR-180LF (TAITEC, Saitama, Japan). Aliquots were stained by 2% sodium phosphotungstate and put on 300-mesh copper girds. The fibrils were imaged using electron microscopy (HT7700, HITACHI, Tokyo, Japan).

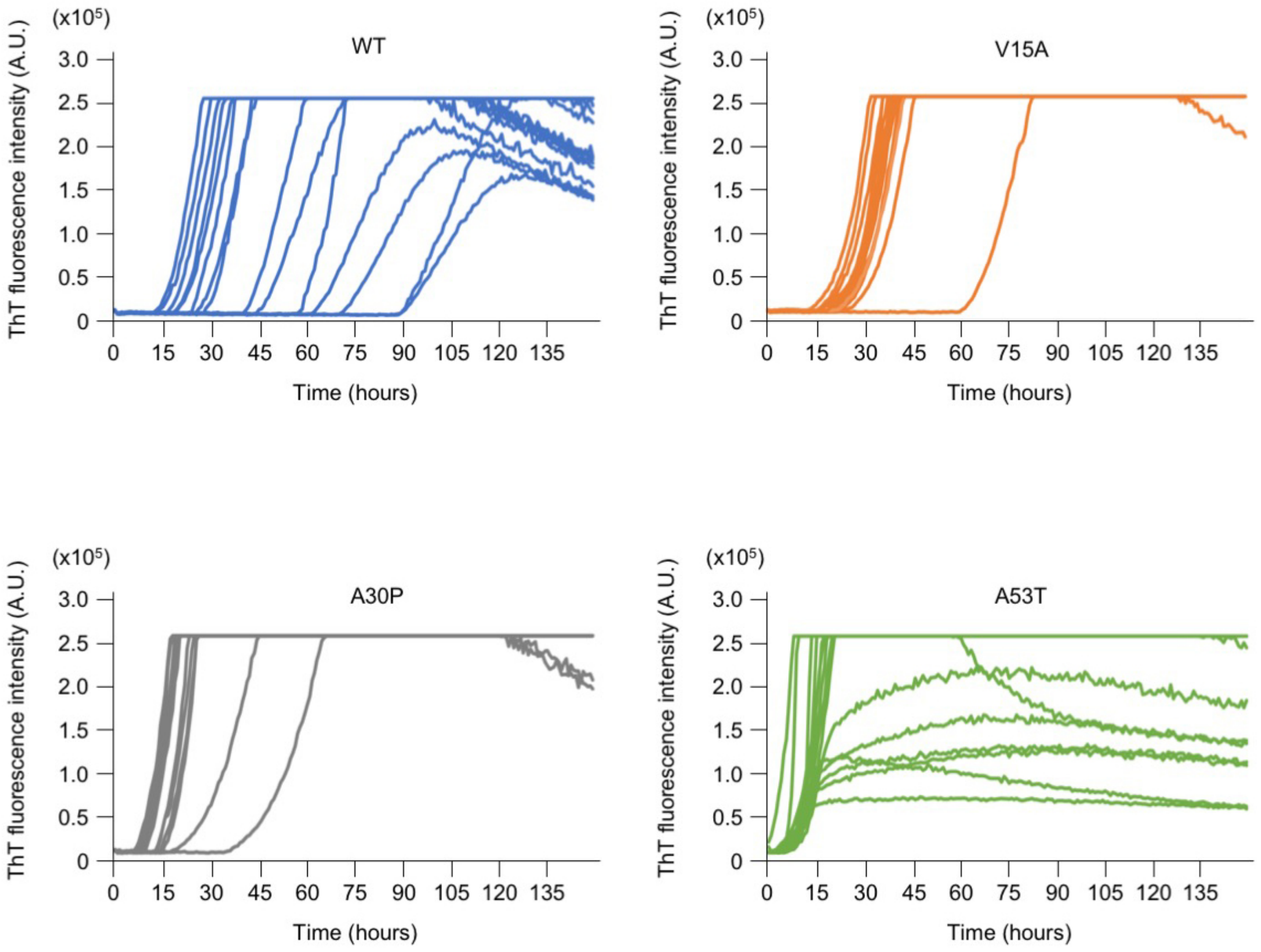

Supporting Information Fig. S1. RT-QUIC amplification profiles of each sample in Fig. 3B.

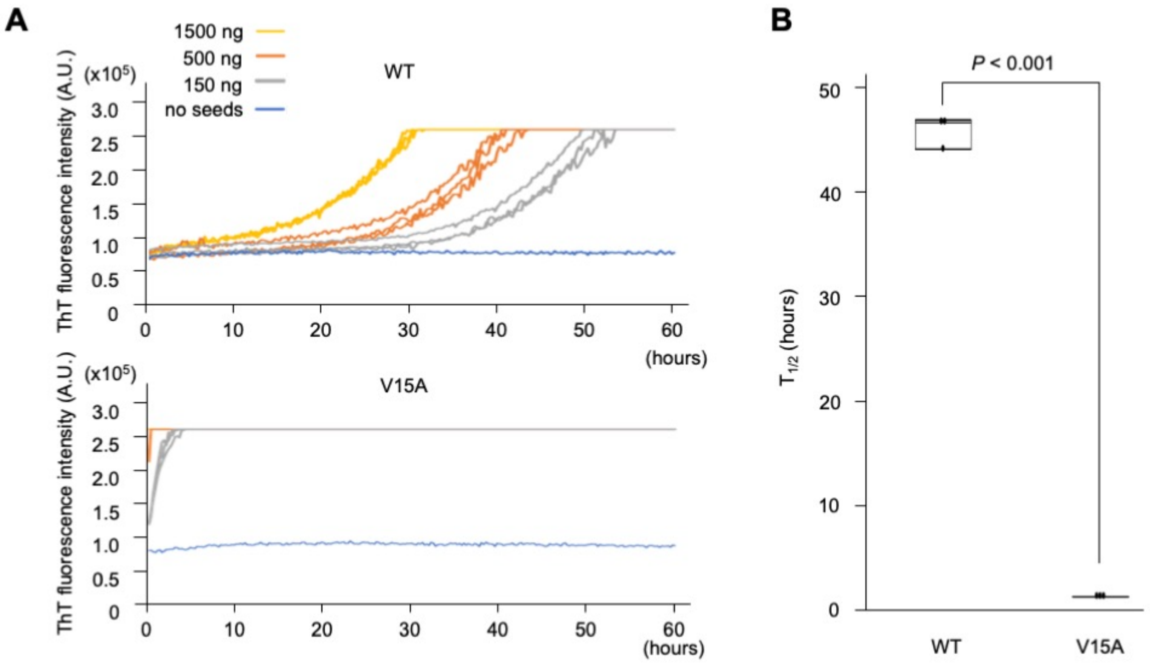

Supporting Information Fig. S2. α-Syn V15A fibrils exhibit a higher seeding activity than WT fibrils *in vitro*. (**A**) RT-QUIC amplification profiles of each sample (seeds, n = 3 each; no seeds, n = 1). (**B**) Graph showing the time when TdT fluorescence reached an intensity of 189,000 RFU ($T_{1/2}$) using 150 ng seeds. $p < 0.001$ calculated by two-tailed Student's t-test.

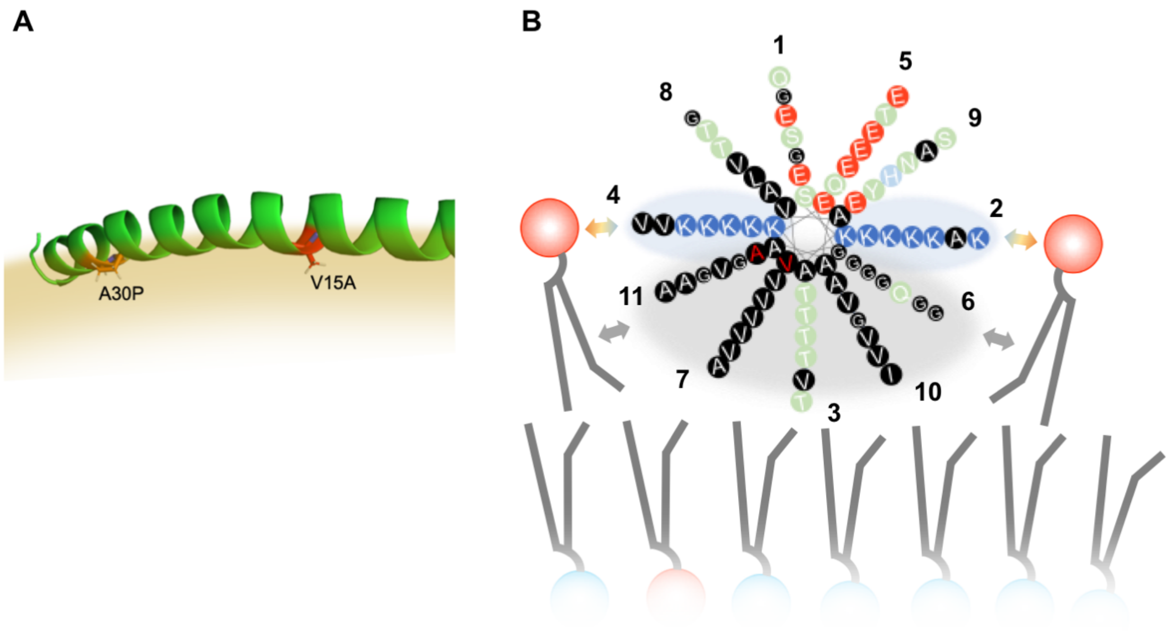

Supporting Information Fig. S3. (**A**) Model of the interface between the first amphipathic helix of α-Syn (green) containing V15 and A30 and phospholipids (light brown). V15 and A30 represent the amino acid side chains when substituted for A and P, respectively. (**B**) The helical wheel (11/3 helix: 3 turns over 11 residues) of a membrane-induced amphipathic helix of α-Syn and its interaction with acidic phospholipids in lipid bilayer membrane. Blue indicates basic, red: acidic, green: polar uncharged, and black: non-polar residues. Red/blue double arrows indicate electrostatic interactions between lysine residues and negatively charged phospholipid head-groups. Gray double arrows indicate hydrophobic interactions between hydrophobic residues and phospholipid acyl-chains. Affected V15 and A30 residues are shown in red. The design of the wheel diagram is adapted from refs.[11,12]

Supporting Table S1. A panel of 16 microsatellites used in haplotype analysis.

Primer name	Size (bp)	Dye	Primer seq. (Forward) （5’→ 3')	Primer seq. (Reverse) （5’→ 3')
D4S3042	179-231	NED	AGCTAACTACTCTCCACCCATAC	CCATGCTAAGTTTATGATGTCTG
D4S2964	159-197	FAM	AAGCTAAGACCCAACTTCTTT	TCATGCAATCCACACAG
D4S2361	149-164	NED	CCACGTGACTTTCATTAGGG	ACACCATCATGGCGCATG
D4S2460	157-191	VIC	CCAAAATCATGTGAGCCA	GAGCAGCAGCCAACTGTAT
D4S2371	185	FAM	ACCAGGGCATTACAATTTGA	TCATGCAGTTGGAAATTTCA
TA46	110-130	VIC	TGTTTGCTACGACATCTCTC	CTTGAGCCAGAAGGTTGAGG
D4S2461	175-185	NED	TACATTATGTTCACACAGTGCCAAG	TTGATAAGAGGCTTGCATTTCC
D4S3475	200-222	FAM	GCACATTCCAGAGCCTTG	AACGAGAGTCTCACTGGGAAC
D4S3477	250-258	FAM	TGGCAGTGTTCTATCAGTGTTG	TCCACTTCACCCAGAATTCC
D4S3480	206-236	VIC	TCCAGCCTGGCAACAGAGT	CCCTGGTCATGACTCTGACACTA
D4S3479	159-173	FAM	TTAACTGCAGAGCACTAACGT	GGGAAGACCAAGCAGATTTC
D4S3474	185-209	NED	AAGTAAGAGTGGACACAAACCTAG	TCACCATTCCAGTTACCATAAC
D4S1544	243-251	FAM	CCATACTAACACAATGGATATAGC	CAGAAACTCCAGCAGAGACT
D4S1089	117-133	NED	TTTTATGCTACACATAATCATG	GGCAAATAAATCGATAGAGGA
D4S414	227-242	NED	TCTTGCACAAAGCATCAGCCCTC	TCAGGAACCTCAGCCCATTTAAG
D4S2380	193-224	FAM	ACCTTATTGTGCTATGGAAACA	TCAGCCAAGATTACCTACCG

Supporting Table S2. The primers for mutagenesis of α-Syn plasmids.

	Forward	Reverse
**SNCA V15A**	5'-GGCCAAGGAGGGAGCTGTGGCTGCTGCTG	5'-CAGCAGCAGCCACAGCTCCCTCCTTGGCC
**SNCA A30P**	5'-GTGTGGCAGAAGCACCAGGAAAGACAAAAG	5'-CTTTTGTCTTTCCTGGTGCTTCTGCCACAC
**SNCA A53T**	5'-GTGGTGCATGGTGTGACAACAGTGGCTGAG	5'-CTCAGCCACTGTTGTCACACCATGCACCAC

Supporting Table S3. ***SNCA*** **V15A evaluated by public genetic databases.**

SNCA	grch37	grch38	SIFT	polyphen2	mutation taster	GEM-J WGA	Tommo14 KJPN	gnomAD v3.1.1 Total	gnomAD v3.1.1 European non-Finnish	gnomAD v3.1.1 East Asian
c.44A>G :p.V15A	chr4:90 756775	chr4:89 835624	0.02	0.987	Disease Causing	NA	NA	0.00000658	0.00000658	0.0

SIFT, Sorting Intolerant From Tolerant; PolyPhen-2, Polymorphism Phenotyping v2; GEM-J WGA, Genome Medical Alliance Japan Whole-genome Aggregation; NA, not available; Tommo, Tohoku Medical Megabank Organization; gnomAD, The Genome Aggregation Database.

Supporting Table S4. Haplotype analysis of family A and family B with *SNCA* c.44T>C (p.V15A).

			Family A								Family B	
Primer name	Region (GRch37/hg19)	Band	III-10		III-11		II-10		III-12		III-1	
D4S3042	76905864-76906247	4q21.1	**234**	217	**234**	219	**234**	230	217	230	217	225
D4S2964	80775313-80775625	4q21.21	**182**	182	**182**	186	**182**	186	182	186	**182**	186
D4S2361	85005386-85005666	4q21.23	**163**	160	**163**	160	**163**	169	160	169	**163**	160
D4S2460	89833649-89834012	4q22.1	**192**	192	**192**	192	**192**	190	192	190	**192**	192
D4S2371	90132775-90133117	4q22.1	**196**	192	**196**	200	**196**	188	192	188	**196**	192
TA46	90191662-90191792	4q22.1	**125**	141	**125**	125	**125**	137	141	137	**125**	125
D4S2461	90232591-90232998	4q22.1	**180**	180	**180**	186	**180**	186	180	186	**180**	186
D4S3475	90668229-90668249	4q22.1	**218**	222	**218**	218	**218**	226	222	226	**218**	218
D4S3477	90709144-90709163	4q22.1	**261**	263	**261**	261	**261**	259	263	259	**261**	263
D4S3480	90713288-90713310	4q22.1	**218**	236	**218**	218	**218**	227	236	227	**218**	236
D4S3479	90730584-90730603	4q22.1	**178**	174	**178**	176	**178**	178	174	178	**178**	172
c.44T>C	90756775		**C**	T	**C**	T	**C**	T	T	T	**C**	T
D4S3474	90785879-90785900	4q22.1	**205**	213	**205**	205	**205**	209	213	209	**205**	207
D4S1544	91028635-91028962	4q22.1	**254**	250	**254**	254	**254**	250	250	250	**254**	254
D4S1089	91736608-91736723	4q22.1	**122**	132	**122**	122	**122**	132	132	132	**122**	136
D4S414	92438661-92439029	4q22.1	**245**	245	**245**	240	**245**	233	245	233	**245**	241
D4S2380	95883055-95883364	4q22.3	220	212	220	220	218	226	220	220	212	212

Supporting Table S5. Clinical summary of PD patients harboring V15A.

Study	Descent	AAO	Segre-gation	Number of the family	Carrier with PD	Carrier without PD	Parkin-sonism	Cogni-tive de-cline	Depres-sion and anxiety	Halluci-nations	Dyski-nesia	Response to levodopa	Autonomic dysfunction
**Cali 2019**	NA	59	NA	1	1	1	1 (100)	1 (100)	1 (100)	1 (100)	NA	1 (100)	NA
**Minafra 2020**	Italian	47-50	Yes	1	3	1*	3 (100)	1 (33)	1 (33)	1 (33)	3 (100)	3 (100)	NA
**Our Cases**	Japa-nese	42-59	Yes (one family)	2	3	1	3 (100)	3 (100)	1 (33)	1 (33)	1 (33)	3 (100)	2/3 (66)
**Overall**		51±6.16		4	7	3	7/7 (100)	5/7 (71.4)	3/7 (42.9)	3/7 (42.9)	4/6 (57.1)	7/7 (100)	2/3 (66)

Data expressed as average ± standard deviation. Percentage in parentheses.

*One subject harboring V15A presented with depression and anxiety without motor symptoms of PD.

Supporting Table S6. Clinical summary of PD patients harboring *SNCA* missense mutation.

	N	AAO	AAE	Time from motor onset to death	Tremor	Depression	Cognitive impairment	Psychosis	Urinary dis-turbances	Orthostatic hypotension
**A30P**	5	59.7 ± 4.3	NA	5.2 ± 4.3	NA	0 (0)	1 (20)	0 (0)	0 (0)	0 (0)
**E46K**	9	52.4 ± 12.5	53.8 ± 18.5	7 ± 1.4	1 (11.1)	3 (33.3)	4 (44.4)	0 (0)	3 (33.3)	4 (44.4)
**H50Q**	3	62.3 ± 7.7	64 ± 6.1	9.6 ± 4.0	3 (100)	0 (0)	3 (100)	0 (0)	0 (0)	0 (0)
**G51D**	8	36.0 ± 12.8	40.7 ± 14.7	21.0 ± 13.7	1 (12.5)	3 (37.5)	3 (37.5)	5 (62.5)	1 (12.5)	3 (37.5)
**A53T**	41	45.6 ± 11.4	52.1 ± 12.0	9.2 ± 5.0	4 (9.8)	2 (4.9)	5 (12.2)	0 (0)	4 (9.8)	4 (9.8)
**V15A (This study)**	3	50.0 ± 8.50	59.3 ± 2.31	20*	2 (66.6)	1 (33.3)	3 (100)	0 (0)	1 (33.3)	1 (33.3)

Data expressed as average ± standard deviation. Percentage in parentheses. Some of the data were referred to ref.[13]

N; number of patients, AAO; age at onset, AAE; age at examination.

*Data from only one patient.

References in Supporting Information

1. Karczewski KJ, Francioli LC, Tiao G, et al. The mutational constraint spectrum quantified from variation in 141,456 humans. Nature 2020;581(7809):434-443.

2. Tadaka S, Hishinuma E, Komaki S, et al. jMorp updates in 2020: large enhancement of multi-omics data resources on the general Japanese population. Nucleic Acids Res 2021;49(D1):D536-D544.

3. Adzhubei IA, Schmidt S, Peshkin L, et al. A method and server for predicting damaging missense mutations. Nat Methods 2010;7(4):248-249.

4. Vaser R, Adusumalli S, Leng SN, Sikic M, Ng PC. SIFT missense predictions for genomes. Nat Protoc 2016;11(1):1-9.

5. Schwarz JM, Rodelsperger C, Schuelke M, Seelow D. MutationTaster evaluates disease-causing potential of sequence alterations. Nat Methods 2010;7(8):575-576.

6. Choi W, Zibaee S, Jakes R, et al. Mutation E46K increases phospholipid binding and assembly into filaments of human alpha-synuclein. FEBS Lett 2004;576(3):363-368.

7. Perrin RJ, Woods WS, Clayton DF, George JM. Interaction of human alpha-Synuclein and Parkinson's disease variants with phospholipids. Structural analysis using site-directed mutagenesis. J Biol Chem 2000;275(44):34393-34398.

8. Fortin DL, Troyer MD, Nakamura K, Kubo S, Anthony MD, Edwards RH. Lipid rafts mediate the synaptic localization of alpha-synuclein. J Neurosci 2004;24(30):6715-6723.

9. Ogata J, Takemoto D, Shimonaka S, Imai Y, Hattori N. α-Synucleinα-synuclein Seeding Assay Using Cultured Cells. In: Imai Y, ed. Experimental Models of Parkinson’s Disease. New York, NY: Springer US, 2021:27-39.

10. Rossi M, Candelise N, Baiardi S, et al. Ultrasensitive RT-QuIC assay with high sensitivity and specificity for Lewy body-associated synucleinopathies. Acta Neuropathol 2020;140(1):49-62.

11. Bendor JT, Logan TP, Edwards RH. The function of alpha-synuclein. Neuron 2013;79(6):1044-1066.

12. Dettmer U. Rationally Designed Variants of alpha-Synuclein Illuminate Its in vivo Structural Properties in Health and Disease. Front Neurosci 2018;12:623.

13. Tambasco N, Nigro P, Romoli M, Prontera P, Simoni S, Calabresi P. A53T in a parkinsonian family: a clinical update of the SNCA phenotypes. J Neural Transm (Vienna) 2016;123(11):1301-1307.
